# Amplifying Older Aboriginal and Torres Strait Islander Women’s Perspectives to Promote Digital Health Equity: Co-Designed Qualitative Study

**DOI:** 10.2196/50584

**Published:** 2023-10-17

**Authors:** Connie Henson, Felicity Chapman, Gina Shepherd, Bronwyn Carlson, Boe Rambaldini, Kylie Gwynne

**Affiliations:** 1 Djurali Centre for Aboriginal and Torres Strait Islander Research and Education Macquarie University Australia; 2 Department of Health Sciences Faculty of Medicine, Health and Human Sciences Macquarie University Macquarie University Australia; 3 Centre for Global Indigenous Futures Macquarie University Macquarie University Australia; 4 Department of Indigenous Studies Faculty of Arts Macquarie University Macquarie University Australia

**Keywords:** digital health, Aboriginal and Torres Strait Islander, Indigenous, health technology, co-design, cultural safety, older Indigenous women, social media, wearables, mobile phone

## Abstract

**Background:**

Digital health is becoming ubiquitous, and we must ensure equity in access. Indigenous people across most high-income countries typically have not benefited as much as other citizens from usual health care systems and technologies. Despite Aboriginal and Torres Strait Islander people’s clear interest in, and enthusiastic use of, new technologies, little research has examined the needs or interests of older Aboriginal and Torres Strait Islander women.

**Objective:**

This study prioritizes the perspectives of older Aboriginal and Torres Strait Islander women, tapping into their expertise associated with Indigenous ways of knowing, being, and doing, as well as their unique position within their families and communities, to design a model for using digital technologies to improve health for themselves and their families as well as their communities.

**Methods:**

Older Aboriginal and Torres Strait Islander women from 4 partner organizations were recruited for this study. This co-designed qualitative research included citizen scientists in shaping the protocol as well as collecting, analyzing, and interpreting data. We used yarning, an Indigenous research method validated for use in health research with Indigenous people and seen as respectful and culturally safe, as a primary research tool. The use of Indigenous methodologies and our iterative process enabled us to deeply explore and incorporate perspectives from all participants and ensure that the perspectives of Indigenous citizen scientists with lived experience were privileged. The data-checking methods also used a yarning methodology, which ensured that the findings and translational model derived from the findings were validated by the participants.

**Results:**

Participants comprised 24 Aboriginal and Torres Strait Islander women aged ≥41 years and including 3 generations that did not grow up with the internet: seniors, baby boomers, and Generation X. The key findings in this research were that older women use various digital technologies to improve health and well-being for themselves and their families as well as their communities. Older Aboriginal women want a culturally sensitive cyberspace that caters specifically to their needs and includes relevant content and functionality that are accessible and efficient. Our translational model highlights the conditions necessary for anyone to use digital health technologies, summarizes the essential elements needed to promote equity in digital health, and illuminates the unmet needs and requirements for older Aboriginal and Torres Strait Islander women to fully benefit from digital health technologies.

**Conclusions:**

Health is a fundamental right. As we move toward greater reliance on digital health solutions, we must recognize and address the concerns of the smaller populations of people who differ in their needs. We must urgently address the financial, connectivity, and other limiting factors highlighted by older Aboriginal and Torres Strait Islander women in this study that limit equitable access to digital health tools.

**International Registered Report Identifier (IRRID):**

RR2-10.1177/20552076221084469

## Introduction

### Background

The proliferation of digital health technologies is revolutionizing how we care for our health [1-3]. From internet searches to social media to wearables, individual users can access general and personalized health information, gather the perspectives of people they trust, and detect and monitor the symptoms and markers of good health. These technologies are already augmenting usual primary and specialist health care [4,5] and have the potential to enhance autonomy and personal accountability for health significantly. These benefits must be equitably distributed.

Indigenous people across most high-income countries typically have not benefited as much as other citizens from usual health care systems and technologies [6,7]; for example, Aboriginal and Torres Strait Islander people in Australia die from avoidable causes at 3 times the rate of other Australians [8]. New health policies, procedures, medicines and technologies are habitually designed and implemented with little or no input from Indigenous people. However, the evidence is that the integration of culture [9-12], having a voice in what and how health care is implemented [13-15], and equitable access that is free of racism and marginalization [16-18] are essential for Indigenous people to experience effective health care in a Western health system. The shift to digital health provides an opportunity to proactively include Indigenous people’s perspectives, needs, and wishes, increasing the equity and effectiveness of health systems.

Innovation and the creation of new technologies have historically been a part of many Indigenous cultural practices and ways that guarantee survival [19]; for example, Brewarrina in New South Wales, Australia, is the home of Baiame’s Ngunnhu (Brewarrina Fish Traps), the oldest human-made structure in the world [20,21]. In modern times, Aboriginal and Torres Strait Islanders are early and avid technology users. As early as 2006, Taylor [22] found that 60% to 80% of the residents aged >10 years in 3 small remote communities in Australia’s Northern Territory owned and regularly used mobile phones despite poor mobile coverage. Likewise, Indigenous people have quickly adapted social media to facilitate connections locally and globally [23]. Innovation with new technologies is also a part of contemporary Indigenous people’s ways of being: Carlson and Dreher [23] noted innovation in using social media for political purposes as well as for empowerment. Aboriginal and Torres Strait Islander people also use and adapt technologies to meet health care needs; for example, Carlson et al [24] examined the role of social media in help seeking and identified 5 types of help seeking related to health and well-being: soliciting support and information, joining health-related groups, using direct messaging, sharing inspiring content, and seeking formal sources of health information. Likewise, Hefler et al [25] found that Aboriginal and Torres Strait Islander adults address various health issues, including mental health and smoking, using eHealth tools. The early adoption of health technologies has occurred not only at the individual user level but also at the organizational level; for example, the Aboriginal and Torres Strait Islander health sector was an early adopter of social media for public health and community development as well as health promotion and advocacy [26].

Despite Aboriginal and Torres Strait Islanders peoples’ clear interest in, and enthusiastic use of, new technologies, little research has examined the needs or interests of older Aboriginal and Torres Strait Islander women. A recent systematic review found that older Indigenous women across high-income countries have been neglected in digital health research. This review identified only 3 papers that focused on the needs and interests of older Indigenous women [27]. This is unfortunate because, in Australia, older Aboriginal and Torres Strait Islander women, like Indigenous women in other high-income countries, have different life experiences, responsibilities, opportunities, and strengths than other people and therefore need to have their perspective illuminated and amplified when new health policies and methods are being designed and implemented.

Likewise, this oversight is inequitable because older Indigenous women in high-income countries are at a higher risk of chronic disease and cancers because of their gender, age, and the negative impacts of racism and colonization [28-31].

Older Aboriginal and Torres Strait Islander women have historically played, and currently play, vital roles in transmitting cultural knowledge and tradition [32]. They have adapted to modern times and continue to invest time and effort to improve the health and well-being of their families and broader communities, including building resilience, educating and supporting young people, role modeling, and helping to shape identity [33]. Older women and Elders also safeguard identity, care for youth, build community resources, pass down knowledge, influence community relations and intergenerational connectedness, and deal with racism [34]. Elders and older Aboriginal and Torres Strait Islander people are respected and trusted. Aboriginal and Torres Strait Islander women often take on cultural leadership roles within their communities [32,33,35]. Their leadership has extended recently to include becoming Indigenous influencers on social media [36], which positions them to provide unique perspectives about how new digital health technologies might be safely and effectively implemented. However, no research has specifically partnered with older Aboriginal and Torres Strait Islander women to address these issues.

This study addresses that gap by prioritizing the perspectives of older Aboriginal and Torres Strait Islander women, tapping into their expertise associated with Indigenous ways of knowing, being, and doing, as well as their unique position within their families and communities, to design a model for using digital technologies to improve health for themselves and their families as well as their communities.

### Aims

The aims of this study were as follows:

1. Identify how older Aboriginal and Torres Strait Islander women use digital health technologies to enhance health.
2. Illuminate case studies of how older women have used digital technologies to enhance health.
3. Develop a working model to inform the development and implementation of digital health technologies that are acceptable and useful for older Aboriginal and Torres Strait Islander women.

We have developed a statement about our use of language related to gender and age, and this is provided at the end of the *Methods* section.

## Methods

A protocol for this study was published in 2022 [37].

### Ethical Considerations

This qualitative translational research adhered to the guidelines for ethical research in Aboriginal and Torres Strait Islander populations and relevant ethics committee approvals. We obtained approval from the Aboriginal Health and Medical Research Council Ethics Committee (1862/21; December 1, 2021).

The consent process was co-designed with the Aboriginal Project Governance (APG) group (for details of this group, refer to the next subsection), and participants had the opportunity to ask questions and consult with others before signing a written (web based or paper) consent form. All participants consented verbally and in writing. Participants were all made aware that they were free to withdraw participation at any point without negative consequences. Participants were given a voucher worth Aus $25 (US $16) as an honorarium to thank them for their time.

### Aboriginal Governance

There is strong evidence that Aboriginal and Torres Strait Islander governance is necessary to ensure a culturally informed research design [38,39]; however, health research methodology has historically minimized or excluded Indigenous ways of thinking, learning, and doing science. Moreover, community priorities have traditionally not informed the direction of health research, resulting in programs that do not meet the needs of Aboriginal people or achieve sustainable outcomes [40]. Since this research project’s inception, the work has been overseen by senior Aboriginal researchers (BC and BR), who are investigators on this study. Two of the 6 authors, including the lead researcher (CH), are non-Indigenous researchers To further empower and amplify Indigenous perspectives, we incorporated Aboriginal and Torres Strait Islander governance into this research by establishing an APG group of citizen scientists to oversee and participate in this study. The APG group comprised older Aboriginal and Torres Strait Islander women who were members of their respective communities. The APG group members agreed upon the terms of reference, which the Aboriginal Health and Medical Research Council Ethics Committee approved.

The APG group members met regularly with the lead researcher to discuss the research. The APG group members consented (written) to record and transcribe these sessions to assist in implementing this project and as part of a separate project to evaluate co-design research in health. In addition to advising on the design of the research protocol, the APG group guided participant recruitment and consent to ensure that participants had a culturally safe and positive experience participating in the research. The APG group also actively advised on data collection and coled the yarning circles for their community members. Two members of the APG group (FC and GS) were actively engaged in the data analysis, reviewing each successive level of analysis and providing insights to ensure that the interpretation of the qualitative data was sensitive to each community’s context, and they are listed as authors on this manuscript. The APG group read and approved this manuscript.

### Rambaldini Model of Collective Impact

**Figure 1:**
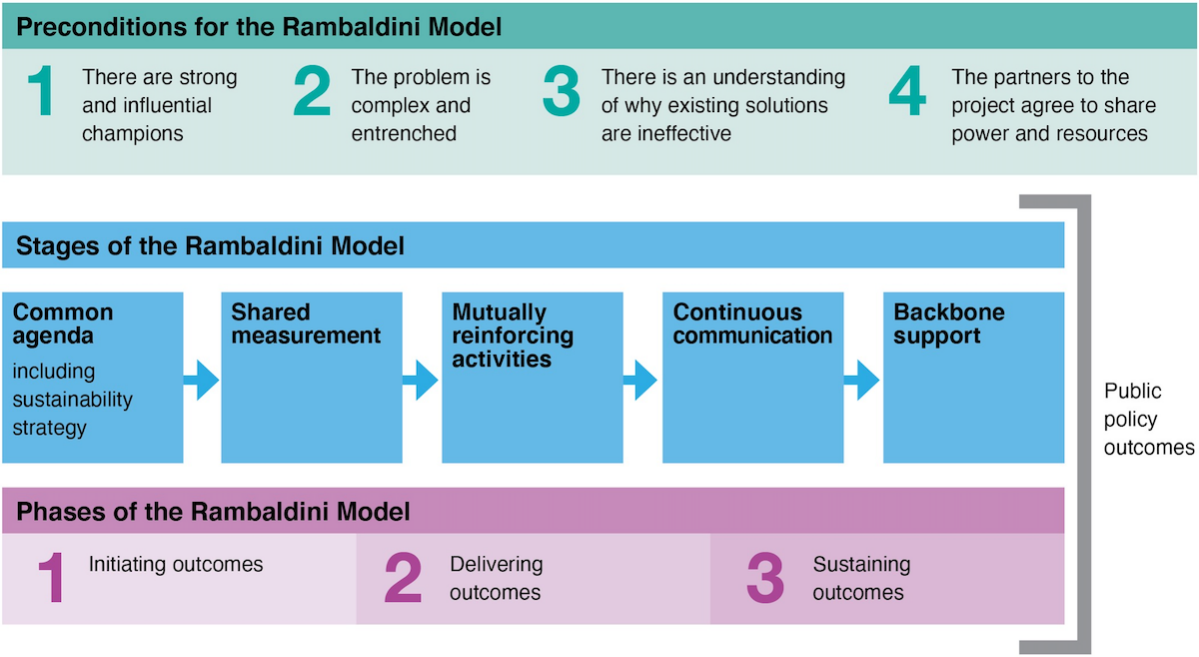
The Rambaldini model.

We used the Rambaldini model of collective impact (Figure 1) to inform our approach to co-design. The Rambaldini model is measurable and structured and requires the sharing of power and resources. The model focuses on the collective rather than the individual, privileges the perspectives of the people affected by the research, and recognizes community contributions in ways that communities experience as meaningful and significant, including coauthorship. The model aligns with the Australian National Health and Medical Research Council guidelines for conducting ethical research with Aboriginal and Torres Strait Islander people [41].

The Rambaldini model aligns with Indigenous ways of knowing, being, and doing and has been validated for health research with Indigenous communities [42]. The model has been used in translational research to improve Indigenous oral health and Indigenous workforce development, as well as to detect and treat atrial fibrillation [43-45].

### Research Communities

Four research communities participated as partners in this research. They included web-based communities of Aboriginal and Torres Strait Islander women who use digital technologies as a regular form of communication. One community was a businesswomen’s network. A second community was a community of women who formed an ecosystem of entrepreneurial women committed to helping each other succeed. A third community was an Aboriginal-led arts and education organization, and the fourth was an Aboriginal and Torres Strait Islander research and education group. As web-based communities, the members included Aboriginal and Torres Strait Islander women who lived in various locations in Australia. Members and leaders from these web-based communities expressed dismay with the limited attention that older Aboriginal and Torres Strait Islander women have received regarding digital health and voiced excitement associated with the potential positive impact that digital technologies can have for older Aboriginal and Torres Strait Islander women.

### Participant Recruitment

The recruitment process was co-designed with the APG group. Using a snowball approach, the APG group members approached women in their communities who they thought would be interested in the research, who in turn recommended other women in their community. We continued to recruit participants until we reached data saturation. The web-based communities were small, and the members were generally known to each other. Women who indicated an interest in participating were provided verbal and written participant information by the APG member.

### Data Collection and Analysis

We used yarning as a key technique to collect, analyze, and interpret data in this research. Yarning is a recognized and validated Indigenous research method for qualitative research, which encourages respectful and honest interactions in a safe place to be heard and respond [46,47]. Our yarning included group yarning circles and one-on-one yarns. The yarning was cofacilitated by an APG group member and the lead investigator. The yarns were prompted with open-ended questions to stimulate discussion. However, the direction and content of the discussions were ultimately determined by participants (Textbox 1).

**Yarning methodology.**

Yarning circles and individual yarns are an Indigenous research tool and a key technique to cocreate knowledge, including collecting, analyzing, and interpreting research data. Yarning can also be used as a co-design technique in research. Yarning is a recognized and validated Indigenous research method for qualitative research [46]. A yarning circle comprises group discussion, whereby facilitators pose questions and invite participants to use the questions as a catalyst for discussion. Similarly, individual yarns are semistructured and begin with questions to stimulate thinking on a particular topic of interest. In the individual and group-based yarns, participants are encouraged to reframe and reinterpret the questions, and the format includes ample time for nonstructured discussion.

The initial questions in the yarn included “What digital technologies do you use for health?” “How do you use these technologies?” We specifically asked about social media, including which platforms they used and whether they gathered health-related information. When relevant to the participant, the yarns also included specific questions related to wearable devices, including “What do you use your wearable device for?” The discussions were facilitated to enable participants to have maximal control over the direction of the discussion, addressing the issues related to digital health that were most relevant to their lives.

Yarning sessions were recorded via Zoom (Zoom Video Communications, Inc) and transcribed. The yarns were cofacilitated by the lead researcher (who is a non-Indigenous researcher) and an APG group member (who is an Indigenous woman). The cofacilitators discussed each session immediately after the session ended. The transcribed sessions were uploaded to NVivo (Lumivero) [48]. The lead author summarized the content of the sessions, highlighting the themes identified by both facilitators immediately after each yarn, adding any additional details from the transcripts, and providing summaries to the APG group for review and discussion until consensus about the key themes was reached. To achieve consensus, the APG group members and lead researcher met weekly to discuss the project and to iteratively incorporate the yarning sessions.

**Figure 2:**
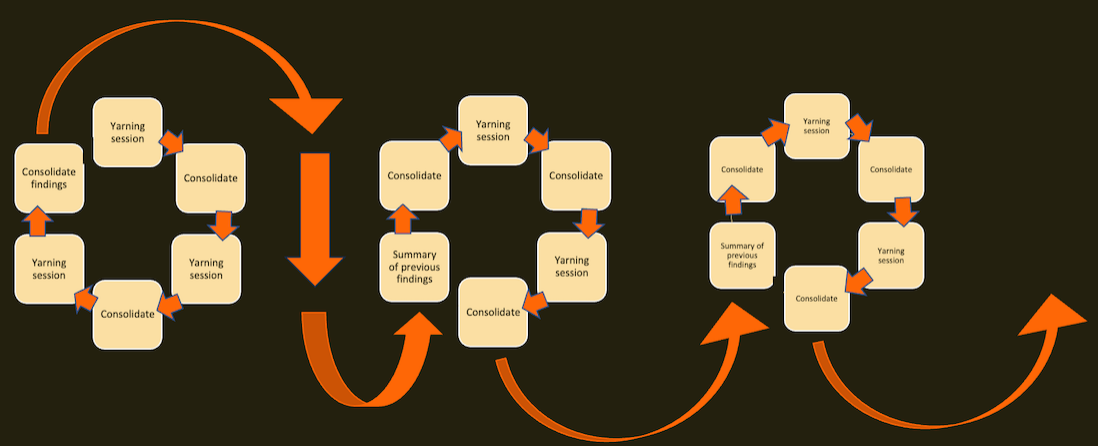
Iterative thinking and interpretation.

Although we were ultimately working toward gaining a consensus, the initial focus of each discussion was to explore different interpretations. Adopting a curious mindset and a yarning communication style ensured that the APG group members and lead researcher deeply understood the thinking, feeling, and experiences informing each person’s views. We privileged the perspectives of the APG group members in all discussions. Privileging was accomplished by the lead researcher asking questions and prompting discussion and APG group members voicing their perspectives and having the final say. The iterative process (Figure 2) enabled the team to incorporate perspectives from all participants and provided multiple opportunities to revise and refine how the group *made sense of the data*, until a consensus was reached on the interpretation and a model that represented the findings. The consolidated findings were then shared with groups of participants for data checking and discussion. The data-checking process included the lead researcher and one of the APG group members meeting with participants via Zoom. These sessions were also conducted in a yarning style and included describing the key findings and asking participants to discuss whether and how each of the findings reflected their perspectives. These data-checking yarns validated the findings and ensured that the content, emphasis, and tone of women’s perspectives were illuminated in the published findings.

### Statement Related to Gender and Age Language in This Study

This research focused on how older Indigenous women use digital health. Our definition of *older* is in keeping with a person’s age when internet use became common. We are therefore defining *older* as generations known as seniors, baby boomers, and Generation X, who did not grow up with the internet [49]. Millennials born after 1980 (aged <41 y) are generally thought to be more comfortable with technology and especially with social media than older generations [50].

Participants in our studies self-identified their gender. Therefore, an Indigenous person who identifies themselves as a woman some or all of the time is included in the studies focused on women. For our studies that include people other than women, we provide various gender options. People are encouraged to self-identify with any description or choose not to describe their gender.

## Results

### Overview

Participants comprised 24 Aboriginal and Torres Strait Islander women from 4 web-based communities. The participants were aged ≥41 years and included 3 generations that did not grow up with the internet: seniors, baby boomers, and Generation X [51].

There were four key findings in the first stage of the study: (1) older Aboriginal and Torres Strait Islander women use technology; (2) there are key barriers; (3) women expect, appreciate, and need cultural sensitivity, relevance, and efficiency from health technologies; and (4) women have unique strengths in their use of technologies to enhance health.

### Finding 1: Older Aboriginal and Torres Strait Islander Women Use Technology for Health and Well-Being

The women described using various technologies, including internet searches, social media, targeting specific websites, web-based presentations, private groups, videos, web-based streaming, Zoom, mobile phone apps, and wearables for health and well-being. They were intentional in how and why they used specific technologies and evolved their use to respond to perceived opportunities or threats. Internet searches were used for first aid, health information they did not want to disclose to others, and validating what their physician told them. Nearly all use social media, especially Facebook. Older technologies, such as static web pages and SMS text messages, were also valued. The women saw social media as a key resource for sharing health information. They were intentional in how they shared information, including targeting specific people for each message. The women who used wearables were avid users, particularly for exercise. Wearables and mobile phone apps were also used to confirm the woman’s beliefs about her health, including sleep and water intake. Some women used their devices to “set their mind at rest.” We highlight these subthemes and illustrate them with quotes from the women in Table 1.

**Table 1:** Technologies for improving health and well-being.

Subtheme	Older Aboriginal and Torres Strait Islander women’s voices
Internet searches most used technology	“Google, like – like everybody in the world, we all just love Google. You know, it’s like – it’s just the best thing.” [Participant 5] “...good translator from the doctor jargon to what we understand when we get home, because you just don’t digest it, and you’ll go home and Google a word...or something...just see what they were talking about.”[Participant 18] “Because sometimes you don’t want to say to the doctor, ‘Well, what does that mean?’...you’ll jump on your phone and have a look.” [Participant 18]
Facebook, YouTube, and Instagram most frequently used social media platforms	“I think, right now, for our generation, we are probably one of the biggest users of Facebook, because it’s more of a connecting type thing.” [Participant 14] “I was glued to Facebook...getting those updates, finding out what’s going on, and even keeping track of World News in how that’s affecting other countries. And, it also gave me the education...reminding me of the, of the face masks...it kind of just empowers that information.” [Participant 10] “...and I think our generation, visual people.” [Participant 10]
Other technologies include old and new media	“I do have a mental health, disability. So I actually use that a lot...for me to make sense of stuff, you know, not, not always Is everything clear?...sometimes you just need that validation.” [Participant 8] “I use technology to do meditation.” [Participant 8] “Now, I don’t carry prescriptions around with me at all...all my prescriptions go straight to the chemist, and I order my scripts online...That to me is like when they invented sliced bread...” [Participant 7]
Targeted and thoughtful sharing health information	“I guess it’s just who might be interested or it’s relevant them.” [Participant 2] “I would say 90% of, of the population, probably even more, but I’m saying 90% of the population, from the ages of eight and up would have a smartphone of some sort...So I would say that would be the most successful way of getting information out into community.” [Participant 2] “It’s the ease. It’s not having to navigate the fandangled words” [Participant 10] “...put messaging on Facebook. And...get trusted Aunties and Uncles to share it.” [Participant 10]
Wearables for activity tracking as a self-therapeutic tool	“I wanted to have a look at what my sleep was at night...Whether or not it’s disturbed or - if I’m actually getting really..., deep sleeps through the night. ‘Cause, quantitively, I feel like I’m a really light sleeper,...I wanted to see, what I look – actually look like - - by using the watch.” [Participant 11] “Well, often, if I’m starting to feel a bit anxious, ‘cause I do suffer anxiety, if I start to feel a bit anxious, I actually check, because it’s something to bring my anxiety down, ‘cause my heart rate usually isn’t up when I’m feeling that anxiety. So, it’s like, you one day, you’re just stress free, you’re over it. It’s like a self-soother. It’s, like, it’s that reality check that brings you back down to earth.” [Participant 4]

### Finding 2: There Are Key Barriers Affecting Older Aboriginal and Torres Strait Islander Women’s Use of Health Technologies

The participants were keenly aware of significant barriers for themselves and other older Aboriginal and Torres Strait Islander women. Even regular users experienced barriers or limiters, including time and emotional burdens, inaccessible language, feeling pressure to respond to others’ needs, racism, and lateral violence; in addition, they could not always find content that met specific needs. The participants also identified practical barriers faced by other Aboriginal and Torres Strait Islander people, including lack of access owing to financial reasons or technical connectivity constraints owing to living remotely:

I think there is a big assumption that a lot of families do have internet when they don’t.

Participant 6

One is that social media has risks and benefits for Aboriginal people. There are some real risks…the racism that’s there, but also the lateral violence.

Participant 14

Use illustrations to explain how things work...basic English—This is what’s happening to your body.

Participant 10

### Finding 3: Older Aboriginal and Torres Strait Islander Women Expect, Appreciate, and Need Cultural Sensitivity, Relevance, and Efficiency From Health Technologies

A central theme that emerged was that women want safe, private cyberspaces designed specifically for Indigenous women that include professional health information, advice from other older Indigenous women users, and the presentation of traditional health and medicine alongside Western approaches. The women want practical content and functionality relevant to their age, gender, and Indigeneity, provided using accessible language and illustrations. The critical needs are summarized and illuminated with quotes from the women in Table 2. The women knew that apps for Indigenous men and boys were available but had not seen anything specific to their needs.

**Table 2:** What women want from technology.

Subtheme	Older Aboriginal and Torres Strait Islander women’s voices
Safe	“...there’s no tracing on what videos they’ve seen and content. Yes...women are going through domestic violence or children going through abuse or anything like that...perpetrators can’t see what content they’ve been watching or downloaded.” [Participant 6] “...verifying whether they’re Indigenous businesses and whether the content they’re sharing is culturally safe.” [Participant 2]
Practical relevant content and functionality is critical	“And I don’t like to have to see something and then I’ve got to, you know, do there’s 10 steps to just share it or save that link.” [Participant 5] “I find government websites are the hardest - like you Google, it gives you a link. And then it takes you to this really generic page.” [Participant 6] “So it’s like quite a specific useful piece of information. It’s like what age can I begin screening? Which age should I begin screening Pap smears. I’m now that I am 51 I like information on what to expect now that I’m in that age group.” [Participant 6] “basic English ‘this is what’s happening to your body.’” [Participant 8]
Culturally sensitive, private web-based spaces for Indigenous women	“First Nations woman to another First Nations woman, that communication is very different to a First Nations woman to a First Nations man. Yeah. you know, and how they speak and understanding and, you know, just context around it.” [Participant 14] “Anything to do with sexual health. Definitely.” [Participant 6] “...grew up with the women being separated from the men. We have women’s business that included intel that the men are not allowed to know about and it’ll be good if we could have some sort of health women’s health information.” [Participant 8] “...need a place there for them to have questions and answers, and especially where they have professional answers, and then they’ve also got the sister answers.” [Participant 9] “It would be so good to have like an app where you can go on and you can ask private questions of women’s business.” [Participant 8] “...get an app, you could put all of that into it, you know. Like, you know, somebody asks a question, and then they get a – a professional answer, they get a community answer, and then they get the pictures...there could be Indigenous nurses, Indigenous doctors talking on there.” [Participant 3] “it would be really good to do an Aboriginal meditation. That would just be so amazing...you could have it like an app that has, you know, spiritual yoga and - You know, medicines that can be used.” [Participant 1]

### Finding 4: Older Aboriginal and Torres Strait Islander Women Have Unique Strengths in Using Technologies to Enhance Health

The women shared innovative uses of technology to promote health in their communities. Two case studies—*Technology-assisted lateral love* (Textbox 2) and *Public health citizen activist* (Textbox 3)—illustrate the women’s broad view of health and their ingenuity in using technology to foster good health and well-being.

**Technology-assisted lateral love.**

- Mental health and well-being were central to how women in our study thought about health. They were particularly interested in and shared examples of how they used technologies to promote their own and others’ mental health and well-being, including using wearables, phone apps and social media.
- “I’ve been using LinkedIn a lot more recently only because of that work profile. I’ve been busy in that space. So I’ve been really trying to share that lateral love on LinkedIn, because we don’t normally get that on that platform. So I think subconsciously, I’ve made an effort to share that Black excellence and that lateral love for other people that are being successful at the moment. So promoting, promoting not just myself, but other Indigenous leaders that are doing well in spaces that we haven’t normally been in. So I think, for me, personally, that was one of the things that I’d kind of made an effort to do now. It’s kind of that natural progression. I get up every morning, and I look at my LinkedIn to see if there’s anything that I need to comment on. It was because there was a lot of lateral violence with other Indigenous leaders on LinkedIn. So I personally, I’m not a part of that. But I’m like, Well, how can I not block that out? But how can I kind of counter that and that’s where that lateral love came from.” [Jaynaya Winmar; approved 20 April, 2023]

**Public health citizen activist.**

- When asked about how they used technology to enhance health, in addition to personal and family health, some women focused on public health. Bush fire preparedness, COVID safety, hydration during heat waves, protection from insects, and environmental hazards, including a campaign to prevent a nuclear waste dump in her community.
- “So the council had a community meeting, and at that community meeting they told us about – I think there were six wonderful things, what they’ve done. It’s like, ‘Wow. This is really, really good.’ Then the next one, it was about nuclear waste. They wanted to build a nuclear waste dump in our community and it was going to bring in millions of dollars. It was going to be $21 million a community gets, and they were going to be able to do this, this and this with it. We didn’t know what to say or think because the only time we knew anything about nuclear was up in The Top End areas and, you know, over in WA and South Australia, and in Sydney we were a little bit shocked. Then, a couple of days after the meeting some bloke came up to me, and he says, ‘Trish, do you realise if they ever build a nuclear waste dump here in Bre, - Bre is only known worldwide for the oldest manmade structure in the world with the fish traps. But if they build a nuclear waste dump nobody will ever know about the fish traps. They’ll only know about the nuclear waste dump.’ And, to me, that was the biggest cultural shock I think I’ve ever had in my entire life. And so, I thought, ‘Oh, well, I’ll stop this.’ I didn’t know how to stop it. I’ve never done a campaign like that before. And, it was through social media that I really got out there. I started up a Facebook page and I put some really good stuff up there. I educated the whole community first, then I educated the outside of the community by saying ‘We need your help. Come and support us.’ Anyway, I prevented it from happening. It was mainly through social media that I was able to do it. …In the beginning, the council would have had about 90 per cent support to build the nuclear waste dump. So, I was able to switch that around by educating the community. And really, most of it was through social media.” [Aunty Trish Frail; approved 20 April, 2023]

In the second stage of the study, we used the 4 findings to cocreate a working model to inform the development and implementation of digital health technologies that are acceptable and useful for older Aboriginal and Torres Strait Islander women.

Taken together, these findings illuminate a path toward equitable digital health.

**Figure 3:**
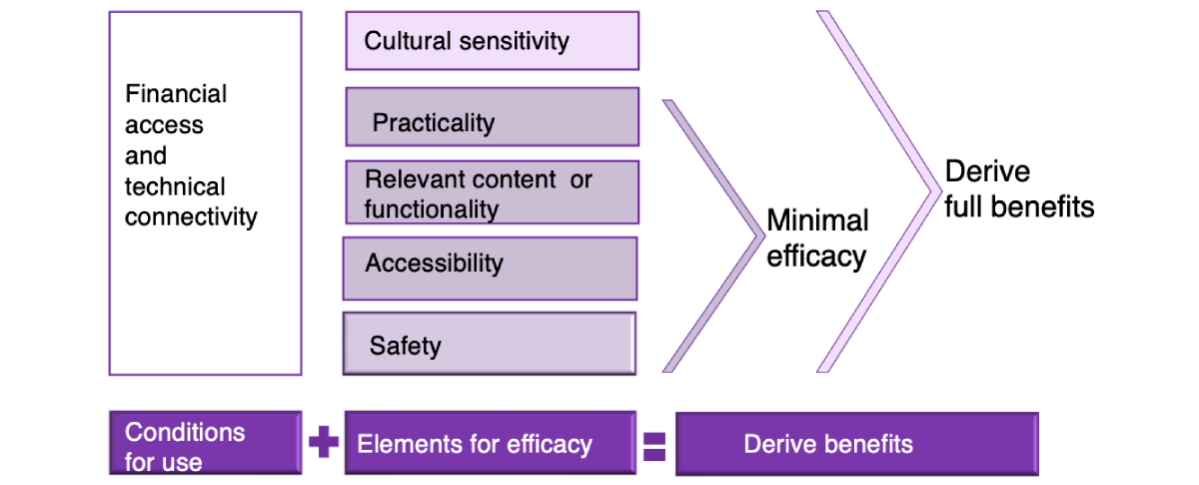
The Djurali model for digital health equity.

The Djurali model for digital health equity (Figure 3) is a translational model that integrates our findings. The model’s first component highlights the conditions for using digital health technologies (financial access and technical connectivity). The model includes the 4 critical elements that must be in place for minimal efficacy enabling people to benefit from these technologies (safety, accessibility, relevant content or functionality, and practicality). Adding a fifth element (cultural sensitivity), which does not currently exist, would enable older Aboriginal and Torres Strait Islander women to use digital tools to meet their unique needs and derive full benefits from these technologies.

## Discussion

### Principal Findings

The key findings in this research were that older Aboriginal and Torres Strait Islander women use various digital technologies and innovate to improve health and well-being for themselves and their families as well as their communities. Older Aboriginal women want a culturally sensitive cyberspace that caters specifically to their needs and includes relevant content and functionality that are accessible and efficient.

The women in this study were avid users of digital health technologies, most commonly internet searches and social media. It was common to gather general information about health conditions and specific information about their body. In addition to using digital health technologies for themselves, the women used technology to benefit family members, their communities, and Indigenous people more generally. The case studies *Technology-assisted lateral love* and *Public health citizen activist* highlight the women’s innovative mindset and self-initiative to use technology to achieve important health and social goals.

Practicality and efficient functionality were seen as essential. The women were keen to have technologies that addressed their specific needs, including age- and gender-relevant health content that included traditional Aboriginal health and medicine alongside Western medicine. They also wanted web-based health advice and recommendations from others in their communities and from health professionals. Systemic issues, including limited access owing to financial or connectivity constraints, as well as racism and inaccessible language were highlighted as significant barriers.

This study identified a gap in the availability of digital health resources specifically for older Aboriginal and Torres Strait Islander women. Recent programs have addressed needs specific to Indigenous men and boys, and some programs address health concerns more specific to younger women and girls [52-54]. However, the women in our study were unaware of digital health programs designed specifically for older Aboriginal and Torres Strait Islander women. Likewise, very little research has focused on digital health for older Indigenous women in high-income countries [27].

### Comparison With Prior Work

Our co-designed qualitative study privileged the perspectives of older Aboriginal and Torres Strait Islander women to understand how they use and could use digital health technologies. It is the only study to focus exclusively on older Aboriginal and Torres Strait Islander women’s experiences with digital health technologies [27]. The study also examines the barriers that Aboriginal and Torres Strait Islander women encounter. The translational model derived from the findings in this study is broadly consistent with other models of digital health equity by centering digital health equity within the broader concept of health equity and recognizing multiple levels and types of influence [55,56]. The Djurali model, like other models of digital health equity, recognizes that digital health technologies are typically developed with and for the broader population without input from a wide diversity of people and, in particular, not considering the perspectives of those people who are most likely to benefit from the technology, such as people living in nonurban locations or people whose interest in, and relationships with, traditional health services are characterized by less trust and comfort [55-58].

The Djurali model is a co-designed model that privileges the perspectives of older Aboriginal and Torres Strait Islander women, thereby addressing the needs and the strengths resulting from the intersectionality of older Aboriginal and Torres Strait Islander women’s age, gender, and Indigeneity. Likewise, how barriers are framed and solutions considered are filtered through a strengths-based lens; for example, the participants in this study framed the disconnect between the intended health message and the person’s understanding of the meaning as resulting from inaccessible language, a failure to use illustrations to explain health concepts, and impractical solutions, which center the problem with the content creator or the technology developer, rather than health or digital literacy, which centers the problem with the individual. The Djurali model is useful for developers and content creators who wish or are required to design programs that are inclusive of older Aboriginal and Torres Strait Islander women. The focus on creating digital health programs that attend to intersectional factors and are strengths based are likely relevant for other populations; however, additional testing and refinement of the Djurali model are necessary to determine the relevancy for other populations.

### Future Directions

Older Aboriginal and Torres Strait Islander women want their own *space—*cyberspace, which enables them to access age- and gender-relevant content in a culturally sensitive way. This includes a space restricted to older Aboriginal and Torres Strait Islander women—consistent with cultural practices that require privacy to discuss sensitive issues considered appropriate when only older women are present. This health-focused space should include traditional and Western approaches to health and include a broad definition of health that encompasses the body, mind, spirit, family, community, and land. It should also enable web-based communication among members but with options for anonymity.

### Knowledge to Action

This paper presents the Djurali model for digital health equity, a translational model of our findings.

We intend to use this model to amplify the perspectives of older Aboriginal and Torres Strait Islander women about what is essential to make digital health equitable.

We urge local, state, and federal governments to recognize that equitable access to health care is a human right. The inevitable shift to digital health presents an opportunity and obligation to respond to the needs of all citizens proactively by addressing the financial and technical connectivity barriers that prevent some citizens from accessing their basic right to equitable health care. Equity is the fundamental reason to create digital health programs for older Aboriginal and Torres Strait Islander women. Other reasons besides equity include that women routinely use their resources to assist their families and communities, spreading the investment well beyond themselves. This proclivity to help others was demonstrated through the case studies in this study and has been documented in other health research and research in economic development [59].

We also encourage health care providers and health technology developers to hold themselves accountable to create digital health technology and programs that are safe, accessible, relevant, and practical and that cater to the unique needs and requirements of all segments of our population. Developers of digital technologies often include user groups in the design, but, to our knowledge, older Aboriginal and Torres Strait Islander women have not been targeted for participation. This is a gap that should be addressed urgently.

Finally, this study used yarning on Zoom as a key methodology. A closer examination of this methodology is underway to better illuminate the factors that contributed to the effectiveness and to identify any aspects of the methods that need to be refined.

### Limitations and Strengths

The first limitation is the narrow inclusion criteria: women who were members of web-based communities and willing to participate via Zoom were eligible. These women represent a subset of older Aboriginal and Torres Strait Islander women. The web-based nature of this study meant that participants lived in various locations across Australia. Thus, the findings from this study may not be relevant for women who are less technology savvy or have less accessibility owing to financial or connectivity constraints. Although we were aware of this limitation, we still purposefully recruited this sample of women because they represent an important cohort of women who are active in their communities and, based on the case studies described in this study, are attuned to, and have an influence in, their communities. Moreover, equity dictates that this cohort also has the right to be heard and their needs catered to, regardless of the generalizability of the findings. Additional research is needed to address how other cohorts of older Aboriginal and Torres Strait Islander women use health technology.

The first strength of this study is the Indigenous governance, which included citizen scientists as coresearchers with lived experience. The co-design process was strongly influenced by privileging the perspectives of the older Aboriginal women coresearchers. The second strength is the use of the Rambaldini model of co-design. Several components of the Rambaldini model were especially helpful in this study, including the common agenda, shared measurement, and the continuous communications. Leaders and members of the web-based communities had independently identified that older Indigenous women were not well represented in the digital health space and were eager to collaborate to understand better and perhaps consider solutions for remedying it. Likewise, the shared measurement and continuous communication components of the Rambaldini model were particularly relevant for the data analysis and interpretation of this study.

In addition, the creation of the translational model provided a straightforward communication tool that can be used by any of the communities and researchers to advocate for practice and policy changes. The cofacilitation led by Aboriginal citizen scientists contributed to cultural safety, from recruitment to data collection, for our participants. Moreover, the active participation of the Aboriginal citizen scientists in the analysis and interpretation as well as the data-checking process we implemented with participants ensured that Aboriginal and Torres Strait Islander perspectives were privileged in interpreting our findings and in our recommendations. The final data-checking yarns validated the data and reinforced findings that the women believed were most critical to communicate through publication. The third strength is that the research design as well as the study were guided by, and evaluated against, the cultural engagement tool based on the criteria set out by the Australian National Health and Medical Research Council’s ethical guidelines for research involving Aboriginal and Torres Strait Islander people [60]. The five criteria are (1) the issue identified by the community, (2) Indigenous governance, (3) capacity building, (4) cultural consideration in the design, and (5) respecting community experience [41,60]. Each of these criteria were met during the course of the research.

### Conclusions

This study sought to identify how older Aboriginal and Torres Strait Islander women use or could use digital health technologies to improve health. The women in this study looked beyond themselves and identified enablers and barriers likely to affect other Indigenous and perhaps non-Indigenous people. They saw access (financial and technical) as a precondition necessary for anyone to use digital health technologies. Moreover, they saw safety, including respectful communication free of racism, as critical. Much more needs to be done to ensure that our web-based spaces are safe and accessible for all people, as noted by a participant:

At the end of the day, deep down inside, everybody’s got to have the same thing. They just want to be treated with respect and consideration, you know.

Participant 13

Digital health is becoming ubiquitous, and we must ensure equity in access. The older Aboriginal and Torres Strait Islander women in this study were proficient in using digital health technologies and keen to be part of the digital revolution. They want technologies that address their unique needs. As we move toward greater reliance on digital health solutions, it is essential that we recognize and address the concerns of the smaller populations of people who differ in their needs. Moreover, we must urgently address the financial and connectivity factors highlighted by the women in this study that preclude equitable access to digital health tools.

## Abbreviations

APG: Aboriginal Project Governance

## Data Availability

The qualitative data generated and analyzed during this study are not publicly available. Because of the historical and ongoing injustices and misuses of personal information experienced by Aboriginal and Torres Strait Islander people in Australia, a condition of our consent to participate is that only summary and thematic data would be made publicly available.
